# Distinct Mitochondrial Recovery Dynamics Following Taxol Removal in Triple-Negative Breast Cancer Cells

**DOI:** 10.3390/cells15191752

**Published:** 2026-09-26

**Authors:** Jack Kollmar, Mihiar Wannousse, Julia Tchou, Lin Z. Li, He N. Xu

**Affiliations:** 1 Department of Surgery, Perelman School of Medicine, University of Pennsylvania, Philadelphia, PA 19104, USA; jack.kollmar@pennmedicine.upenn.edu (J.K.); mihiar.wannousse@pennmedicine.upenn.edu (M.W.); julia.tchou@pennmedicine.upenn.edu (J.T.); 2 Britton Chance Laboratory of Redox Imaging, Department of Radiology, Perelman School of Medicine, University of Pennsylvania, Philadelphia, PA 19104, USA; linli@pennmedicine.upenn.edu

**Keywords:** optical redox imaging (ORI), NADH, multinucleation, ROS, MMP, drug resistance, metabolic adaptation, recovery-associated metabolic phenotypes

## Abstract

**Highlights:**

**What are the main findings?**

- Mitochondrial and cellular recovery after Taxol withdrawal are dynamic and multidimensional, with distinct recovery trajectories in TNBC cells of differing Taxol sensitivity.
- Persistent mitochondrial redox alterations in MDA-MB-436 cells are accompanied by incomplete viable-cell population recovery and residual multinucleation.

**What are the implications of the main findings?**

- Mitochondrial recovery, resolution of multinucleation, and population recovery occur on different timescales following Taxol withdrawal.
- Longitudinal metabolic imaging may provide a non-destructive approach for identifying persistent post-treatment metabolic states and assessing recovery beyond conventional treatment-endpoint measurements.

**Abstract:**

Drug resistance in TNBC is associated with metabolic plasticity, yet little is known about how mitochondrial and cellular states evolve after chemotherapeutic withdrawal. We used optical redox imaging (ORI) microscopy and complementary functional measurements to characterize post-Taxol recovery in two TNBC cell lines with different Taxol sensitivities. Whole-dish nuclear counting following 48 h Taxol treatment confirmed that HCC1806 cells responded at lower Taxol concentrations than MDA-MB-436 cells. Recovery was subsequently monitored following extracellular Taxol withdrawal using mitochondrial redox indices, mitochondrial reactive oxygen species (mito-ROS), mitochondrial membrane potential (MMP), multinucleation, and live- and dead-cell counts. HCC1806 cells showed substantial recovery of several mitochondrial parameters and resolution of multinucleation by 96 h; however, a late reductive shift in the optical redox ratio (ORR), reduced MMP, and attenuated live-cell population expansion indicated incomplete redox and functional recovery. In contrast, MDA-MB-436 cells exhibited persistent elevations in Fp and ORR through 144 h, residual multinucleation, and little sustained live-cell population expansion. These findings demonstrate that mitochondrial, multinucleation, and population-level recovery can proceed on different timescales and that post-treatment recovery differs markedly between TNBC models with different Taxol sensitivities. Longitudinal ORI provides a non-destructive approach for characterizing persistent post-treatment metabolic states and, together with functional measurements, may facilitate investigation of recovery-associated phenotypes relevant to therapeutic response.

## 1. Introduction

Chemotherapy remains a cornerstone of treatment for triple-negative breast cancer (TNBC), yet acquired and intrinsic resistance continue to limit its long-term efficacy. Growing evidence indicates that metabolic reprogramming contributes to therapeutic resistance, enabling TNBC cells to survive chemotherapy-induced stress through flexible utilization of bioenergetic pathways [1,2,3,4].

Studies have shown that resistant TNBC cells can adopt distinct metabolic states, including increased reliance on oxidative phosphorylation (OXPHOS) or glycolysis [2,4,5]. However, these studies have largely focused on metabolic alterations during drug exposure. Much less is known about how mitochondrial metabolism evolves after drug removal, the extent to which mitochondrial homeostasis is restored, and whether recovery trajectories differ among TNBC cells with different therapeutic sensitivities.

Paclitaxel (Taxol^®^) is a standard chemotherapeutic for treating TNBC. Taxol binds to and stabilizes microtubules, suppressing microtubule dynamics and disrupting normal mitotic progression, which can ultimately lead to cell death [6]. Taxol has also been shown to alter mitochondrial metabolism and redox function [7,8,9]. In TNBC, OXPHOS-dependent subpopulations and cancer stem-like cells with high mitochondrial activity have been associated with chemoresistance [5,10], while residual tumor cells surviving chemotherapy can exhibit metabolically flexible, OXPHOS-high states [11,12]. Conversely, Taxol has also been reported to induce a glycolytic switch in patient-derived TNBC organoids [2], highlighting the metabolic plasticity of TNBC under therapeutic stress. More recently, longitudinal optical metabolic imaging further demonstrated metabolic reprogramming during tumor regression, residual disease, and recurrence [4].

While these studies demonstrate the importance of metabolic adaptation under chemotherapeutic stress, the post-treatment recovery phase remains poorly characterized, particularly in TNBC. It is largely unknown whether mitochondrial and cellular parameters return synchronously to their pretreatment states or follow distinct recovery trajectories. Characterizing this recovery phase may provide additional insight into the fate of cells that survive chemotherapy and reveal post-treatment metabolic phenotypes not captured by conventional treatment-endpoint measurements.

Label-free live-cell optical redox imaging (ORI) assesses mitochondrial redox state through the intrinsic fluorescence of reduced nicotinamide adenine dinucleotide (NADH) and oxidized flavin adenine dinucleotide-containing flavoproteins (Fp) [13,14,15]. These mitochondrial redox coenzymes reflect the balance between electron production through metabolic pathways and electron consumption by the electron transport chain. Changes in their relative abundance provide a functional readout of mitochondrial redox balance. The mitochondrial redox state is often expressed as the optical redox ratio (ORR), Fp/(NADH + Fp), which provides an integrated measure of mitochondrial redox balance. A higher ORR indicates a more oxidized state, whereas a lower ORR indicates a more reduced state. Because ORI is non-destructive, it is particularly suited for investigating dynamic metabolic responses following therapeutic perturbation, as demonstrated in our previous studies of HCC1806 cells showing rapid redox changes during drug exposure [16] and post-treatment redox responses following drug removal [17].

In the present study, we investigated post-Taxol recovery in two TNBC cell lines with different Taxol sensitivities, HCC1806 and MDA-MB-436. Using ORI together with measurements of mitochondrial ROS (mito-ROS), mitochondrial membrane potential (MMP), multinucleation, and live- and dead-cell populations, we characterized mitochondrial and cellular recovery following extracellular Taxol withdrawal. We show that the two cell lines exhibit distinct mitochondrial and functional recovery trajectories, demonstrating that post-treatment recovery is a dynamic and multidimensional process.

## 2. Materials and Methods

### 2.1. Cell Preparation and Treatment

Human TNBC lines HCC1806 and MDA-MB-436 were purchased from ATCC (Manassas, VA, USA). The cells were cultured in RPMI 1640 (Gibco Cat # 11875085, Thermo Fisher Scientific, Waltham, MA, USA) supplemented with 10% fetal bovine serum (FBS) (MilliporeSigma, St. Louis, MO, USA). Cultures were maintained at 37 °C and 5% CO_2_ in T-flasks. For imaging, an appropriate number of cells was seeded in glass-bottom dishes (Cat #. D35-14-1.5-N, Cellvis, Mountain View, CA, USA).

For Taxol treatment and recovery experiments, cells were seeded in glass-bottom dishes containing 2 mL of culture medium. Taxol was prepared in DMSO at the appropriate concentration, and 2 µL was added to each dish (1:1000 dilution). Mock control cells received 2 µL of DMSO (final concentration, 0.1%). Cells were treated with Taxol for 48 h. For recovery experiments, after 48 h of Taxol treatment, the Taxol-containing medium was aspirated, the cells were washed twice with PBS, and fresh drug-free medium was added. This time point was designated 0 h of recovery. The culture medium was replaced every 48 h throughout the recovery period. Cells were assayed immediately after Taxol removal (0 h of recovery) and at the indicated recovery time points thereafter. To prevent cell overgrowth during extended culture, initial seeding densities were adjusted according to the planned recovery duration to maintain comparable cell confluency between treated and control dishes throughout the experiment and to minimize the influence of cell density on metabolic measurements.

Cell growth assays for the recovery trajectory experiments were performed in 24-well plates. At the indicated recovery time points, both floating and adherent cells were collected, stained with trypan blue, and counted using a hemocytometer to quantify live and dead cells. At 0 h of recovery, live Taxol-treated cells were counted, and cells that had undergone 48 h of 0.1% DMSO treatment were seeded into control wells at the same live-cell density, thereby establishing comparable starting viable-cell densities for the subsequent recovery trajectories.

### 2.2. Imaging and Data Processing

Thirty minutes before imaging, cells in the glass-bottom dishes were rinsed twice with phosphate-buffered saline (PBS, Cat.# MT21040CV, Thermo Fisher Scientific, Waltham, MA, USA) and maintained in 1 mL Live Cell Imaging Solution (Cat.# A59688DJ, manufactured in Grand Island, NY, USA, Invitrogen™) supplemented with 11 mM glucose and 2 mM L-glutamine (LCIS^+^) for imaging.

To identify an appropriate Taxol concentration for studying recovery dynamics, the same dishes used for ORI were subsequently stained with Hoechst 33342 (10 µM) and subjected to whole-dish imaging using a 5× objective. Hoechst-positive nuclei were counted across the entire dish using ImageJ (v1.54r), allowing mitochondrial redox responses and population-level changes to be assessed in the same experimental samples.

To detect mitochondrial superoxide, MitoSOX Red (Thermo Fisher Scientific, Waltham, MA, USA) was added to the dishes after ORI at a final concentration of 2 µM; control dishes received the corresponding volume of DMSO. Dishes were then incubated at 37 °C for 10 min in the dark, rinsed once with PBS, and imaged in 1 mL PBS.

To detect mitochondrial membrane potential, TMRE (Thermo Fisher Scientific, Waltham, MA, USA) was added to the dishes after ORI at a final concentration of 200 nM; control dishes received the corresponding volume of DMSO. Dishes were incubated at 37 °C for 20 min in the dark, rinsed once with PBS, and imaged in 1 mL PBS.

ORI acquisition and data processing have been described in detail in our previous works [8]. Briefly, live-cell culture dishes were imaged using a wide-field fluorescence microscope (Zeiss Observer 7, White Plains, NY, USA). 3–10 randomly selected fields of view (FOVs) were captured per dish, and samples were maintained at 37 °C during imaging. Regions of interest (ROI) were located and focused using transmitted light to avoid photobleaching. The vignette effect was corrected on the fly. The imaging settings are listed in Table 1.

The raw images were processed using a customized MATLAB^®^ (Version 2019a, The MathWorks, Inc., Natick, MA, USA) program to first remove the background, then threshold pixel intensities at a signal-to-noise ratio (SNR) of 7.5 to obtain the net intensities of NADH and Fp., ORR images were generated on a pixel-by-pixel basis as Fp/(NADH + Fp), and the mean ORR for each FOV was calculated from the resulting pixel-wise ORR values. An SNR of 5 was applied to obtain signals from the MitoSOX and TMRE fluorescent tracers. The mean values of the imaging metrics from each FOV were averaged to obtain dish means, which were then averaged across all dishes to calculate the group means.

Because mitochondrial NADH autofluorescence delineates the cytoplasm while nuclei appear as dark regions, NADH images were used to quantify the percentage of multinucleated cells by manual counting in ImageJ. Only images containing sufficient numbers of cells to allow reliable discrimination between mononucleated and multinucleated cells were included in the analysis. Biological control dishes were included in all imaging sessions, and data were normalized to the respective control dish mean and expressed as fold change.

### 2.3. Statistical Analysis

For recovery experiments, separate sets of dishes were prepared for each recovery time point; the same dishes were not repeatedly imaged over time. For normalized recovery measurements, comparisons with the respective time-matched controls (fold change = 1) were performed using two-tailed one-sample Student’s *t*-tests. To account for multiple testing across the recovery timeline, *p*-values were adjusted using the Holm–Šídák method within each measured endpoint. Live-cell counts during recovery were analyzed using ordinary two-way ANOVA followed by Šídák’s multiple comparisons test between DMSO control and Taxol-pretreated groups at each time point. Multinucleation across recovery time points was analyzed using ordinary one-way ANOVA followed by Dunnett’s multiple comparisons test versus 0 h. Other group comparisons were analyzed using ordinary one-way ANOVA followed by Dunnett’s multiple comparisons test, as indicated. Statistical analyses were performed using GraphPad Prism (v11). Adjusted *p* < 0.05 was considered statistically significant for analyses involving multiple comparisons.

## 3. Results

### 3.1. Differential and Concentration-Dependent Responses of TNBC Cells to Taxol Treatment

To investigate the metabolic effects of Taxol on TNBC cells, HCC1806 and MDA-MB-436 cells were examined. HCC1806 has been classified as a BL2 cell line, whereas MDA-MB-436 has been classified as mesenchymal-stem-like [18,19,20,21,22]. The two cell lines also exhibited different morphology and mitochondrial redox states (Figure 1). Under control conditions (DMSO = 0.1%, 48 h), the two cell lines exhibited different redox indices, with HCC1806 cells showing lower Fp and NADH but a higher ORR than MDA-MB-436 cells.

To identify Taxol concentrations that produced comparable metabolic and population-level responses in the two cell lines, HCC1806 and MDA-MB-436 cells were treated with increasing concentrations of Taxol for 48 h and subjected to ORI followed by Hoechst 33342 staining and whole-dish nuclear imaging. Representative whole-dish nuclear images are shown in Figure S1. Taxol induced concentration-dependent changes in mitochondrial redox indices (Fp, NADH, ORR) and nuclear counts in both TNBC cell lines, with HCC1806 cells responding at lower concentrations than MDA-MB-436 cells (Figure 2). In HCC1806 cells, Fp and NADH increased significantly beginning at 2 nM Taxol and remained elevated at higher concentrations, whereas ORR showed only modest changes across the concentrations examined (Figure 2A–C). Nuclear counts decreased with increasing Taxol concentration, with an approximately 50% reduction relative to control at 2 nM and little further reduction at higher concentrations (Figure 2D).

MDA-MB-436 cells exhibited a redox response at higher Taxol concentrations. Fp increased significantly with Taxol treatment, whereas NADH showed a modest upward trend that did not reach statistical significance over the concentrations examined (Figure 2E,F). ORR increased significantly at higher Taxol concentrations, indicating an oxidative shift in mitochondrial redox state (Figure 2G). Nuclear counts also decreased in a concentration-dependent manner, reaching an approximately 50% reduction at 10 nM Taxol, with little additional reduction at 20 nM (Figure 2H). Together, these nuclear count data demonstrate that HCC1806 cells respond to lower Taxol concentrations than MDA-MB-436 cells, supporting differential Taxol sensitivity between the two cell lines. Absolute Fp, NADH, ORR, and nuclear counts for both cell lines are provided in Figure S2.

In HCC1806, multinucleation was present in only a few cells at 0 nM and 1 nM, in about 50% of cells at 2 nM, and in nearly all cells at 5 nM and 10 nM Taxol (Figure 3A–F). The redox indices of individual mononucleated and multinucleated cells at 2 nM Taxol showed similar values between the two cell populations (Figure S3A–C).

In MDA-MB-436 cells, the proportion of multinucleated cells was low at baseline and increased with Taxol concentration up to 10 nM, reaching approximately 60% at 10 and 20 nM (Figure 4A–F). As in HCC1806 cells, similar Fp, NADH, and ORR values were found between mononucleated and multinucleated cells following treatment with 20 nM Taxol for 48 h (Figure S3D–F).

### 3.2. Mitochondrial and Cellular Recovery Following Taxol Removal

Next, we investigated mitochondrial recovery following Taxol removal. To enable comparison of recovery between the two cell lines, cells were treated for 48 h with Taxol concentrations of 2 nM for HCC1806 and 10 and 20 nM for MDA-MB-436. These concentrations produced broadly comparable (~25%) changes in Fp and NADH together with substantial multinucleation, as determined from the dose-titration experiments. In addition, 2 nM Taxol in HCC1806 and 10 nM Taxol in MDA-MB-436 produced comparable reductions (~50%) in nuclear counts (Figure 2D,H).

Immediately after Taxol removal (0 h recovery), HCC1806 cells exhibited increases in NADH and Fp (Figure 5A,B). The absolute values of the redox indices of both control and Taxol-pretreatment groups are shown in Figure S4A–C. Compared with the respective time-matched controls, Fp remained significantly elevated through 72 h, whereas NADH remained significantly elevated through 48 h and subsequently approached the time-matched control level (Figure 5A,B). ORR did not differ significantly from control through 72 h but was significantly lower at 96 h (Figure 5C). Mito-ROS fluctuated around the control level without significant differences (Figure 5D), whereas MMP was significantly reduced at 96 h (Figure 5E). Live-cell counts increased during recovery in both groups, but the Taxol-pretreated population showed significantly attenuated expansion over time (treatment × recovery time interaction, *p* = 0.0014), with significantly fewer live cells than the time-matched controls from 48 through 96 h (Figure 5F). The dead-cell percentage approached the control level by 48 h (Figure S5A). Multinucleation progressively declined after Taxol removal and was nearly absent by 96 h (Figure 5G).

Following the removal of 10 nM Taxol treatment, MDA-MB-436 cells showed persistent mitochondrial and cellular alterations (Figure 6). Fp and ORR remained significantly elevated throughout the 144 h recovery period, whereas NADH showed modest fluctuations above the respective control levels (Figure 6A–C). The absolute values of the redox indices of both control and Taxol-pretreatment groups are shown in Figure S4D–F. The persistently elevated ORR indicated a sustained oxidative shift in mitochondrial redox state. Mito-ROS remained above the control level, with the largest mean elevation at 96 h, while MMP showed a marked decrease at 96 h (Figure 6D,E).

Live-cell counts showed markedly different recovery trajectories between DMSO control and Taxol-pretreated cells (treatment × recovery time interaction, F(3,15) = 298.7, *p* < 0.0001). Although initially comparable, live-cell numbers became significantly lower in Taxol-pretreated cultures from 48 h onward, with the DMSO population subsequently expanding substantially while Taxol-pretreated cells showed little sustained population expansion (Figure 6F). Multinucleation remained prevalent during recovery, although the percentage of multinucleated cells decreased significantly by 144 h compared with 0 h (Figure 6G). The proportion of dead cells in Taxol-pretreated cultures decreased during early recovery but increased again at later time points, whereas it remained relatively low in DMSO controls (Figure S5B). Although this late increase did not result in a significant treatment × recovery time interaction, Taxol-pretreated cultures exhibited a significantly higher overall proportion of dead cells than DMSO controls. Together, these findings indicate that MDA-MB-436 cells failed to restore mitochondrial redox homeostasis and showed poor population-level recovery following 10 nM Taxol treatment.

Consistently, following removal of 20 nM Taxol, MDA-MB-436 cells also exhibited persistent redox alterations during the 120 h recovery period (Figure 7). Fp remained significantly elevated throughout recovery, while NADH remained elevated at several time points, including 120 h (Figure 7A,B). ORR remained above the respective control levels throughout recovery, with a significant elevation at 0 h (Figure 7C). Multinucleation persisted, decreasing from approximately 50% at 0 h to approximately 40% at 120 h (Figure 7D). Descriptive FOV-level measurements showed elevated mito-ROS and fluctuations in MMP during recovery (Figure S6).

## 4. Discussion

Our study demonstrates that mitochondrial recovery following Taxol removal is a dynamic and multidimensional process in TNBC cells. While most studies focus on metabolic alterations during drug exposure, our results show that mitochondrial and cellular alterations continue after extracellular drug withdrawal. Longitudinal measurements of mitochondrial redox indices, mito-ROS, MMP, multinucleation, and live-cell population dynamics revealed markedly different recovery trajectories between HCC1806 and MDA-MB-436 cells, indicating that recovery involves continued biological adaptation rather than simply reversal of treatment-induced perturbations.

In HCC1806 cells, Fp and NADH approached their respective time-matched controls during recovery, mito-ROS fluctuated around baseline, and multinucleation largely resolved by 96 h. However, ORR showed a late reductive shift, and MMP remained reduced at 96 h. Live-cell counts also demonstrated incomplete functional recovery: although Taxol-pretreated cells continued to expand, their population trajectory differed significantly from that of controls, with live-cell numbers remaining substantially lower at 96 h. Thus, recovery of several mitochondrial parameters and resolution of multinucleation did not coincide with complete restoration of mitochondrial redox homeostasis or population-level recovery.

MDA-MB-436 cells exhibited a more persistent post-treatment phenotype. Following treatment with 10 nM Taxol, which produced a comparable (~50%) reduction in nuclear counts to that observed with 2 nM Taxol in HCC1806 cells, Fp and ORR remained significantly elevated throughout the 144 h recovery period, whereas NADH, mito-ROS, and MMP showed temporal fluctuations. Residual multinucleation was also observed, and live-cell counts remained low compared with the substantial expansion of the control population. Whether these alterations ultimately resolve beyond 144 h or represent a more persistent phenotype will require longer-term observation. These findings are consistent with a persistently altered metabolic and functional state rather than restoration to the control condition.

The distinct recovery trajectories of HCC1806 and MDA-MB-436 cells may reflect intrinsic differences between the two cell lines in addition to their different Taxol sensitivities. For example, differences in basal mitochondrial redox state, proliferative characteristics, and capacity to restore mitochondrial homeostasis after treatment could influence the observed recovery patterns. Therefore, the divergent recovery trajectories should not be attributed to Taxol sensitivity alone. Conversely, some of these intrinsic properties may themselves contribute to differential Taxol sensitivity.

The persistence of mitochondrial redox alterations after Taxol removal further suggests that the post-treatment period may represent a biologically relevant phase of metabolic adaptation. This interpretation is consistent with the broader concept of metabolic plasticity in TNBC [1,2,3] and with reports that residual tumor cells surviving chemotherapy can develop dependencies on OXPHOS and related mitochondrial pathways [5,9]. Although therapeutic vulnerability during recovery remains to be tested directly, persistent post-treatment redox alterations may provide opportunities for targeting mitochondrial metabolism, redox regulation, or oxidative-stress responses. Longitudinal ORI may provide a useful approach for identifying such altered post-treatment metabolic states and determining whether metabolic recovery corresponds with subsequent functional recovery.

Notably, persistent ORR alterations in both cell lines coincided with incomplete population recovery: HCC1806 cells exhibited a late reductive shift in ORR together with attenuated population expansion, whereas MDA-MB-436 cells maintained an elevated ORR together with little sustained population expansion. Longer-term studies will be needed to determine whether the persistent metabolic alterations ultimately resolve and to further define their relationship with functional recovery.

Several limitations in our study should be considered. ORI-derived NAD(P)H fluorescence does not distinguish NADH from NADPH; therefore, changes in the measured signal may reflect both bioenergetic and antioxidant processes, particularly because NADPH provides reducing equivalents for major cellular antioxidant systems. The study was also limited to two TNBC cell lines, and additional models will be required to determine the generality of the observed recovery phenotypes. Furthermore, ORI identifies mitochondrial redox alterations but does not directly determine the metabolic pathways responsible for them; complementary metabolic flux analyses and targeted perturbations will be needed to establish the underlying mechanisms. Although Taxol concentrations were selected to produce comparable metabolic and population-level perturbations, equivalent biological responses do not necessarily imply equivalent pharmacological exposure, and the recovery differences should therefore be interpreted in the context of each cell line’s intrinsic Taxol sensitivity.

Finally, the experiments characterize recovery following extracellular Taxol withdrawal. Although Taxol-containing medium was removed, the cells were washed, and drug-free medium was added; intracellular Taxol clearance was not measured, so residual intracellular Taxol during recovery cannot be excluded. Previous work has demonstrated intracellular paclitaxel retention following extracellular drug withdrawal [23]. However, the same study showed that delayed effects following 24 h of paclitaxel exposure were independent of the amount of intracellularly retained drug [23].

Collectively, our findings support a model in which recovery following chemotherapy is a dynamic adaptive process that may lead either to partial restoration of mitochondrial homeostasis or to a persistent metabolically altered state associated with incomplete functional recovery, with potential implications for differential therapeutic sensitivity.

## 5. Conclusions

Our study demonstrates that recovery following Taxol treatment is a dynamic and multidimensional process in TNBC cells, involving distinct trajectories of mitochondrial redox status, mitochondrial function, multinucleation, and viable-cell population recovery. HCC1806 and MDA-MB-436 cells exhibited different Taxol sensitivities and markedly different post-treatment recovery characteristics, with mitochondrial and functional alterations persisting after extracellular drug withdrawal. These findings highlight the recovery period as an active component of the chemotherapy response and demonstrate the utility of live-cell optical redox imaging for longitudinally characterizing post-treatment metabolic states. The correspondence between persistent redox alterations and incomplete population-level recovery provides a basis for further investigation of ORI as a non-destructive indicator of post-treatment cellular states.

## Figures and Tables

**Figure 1:**
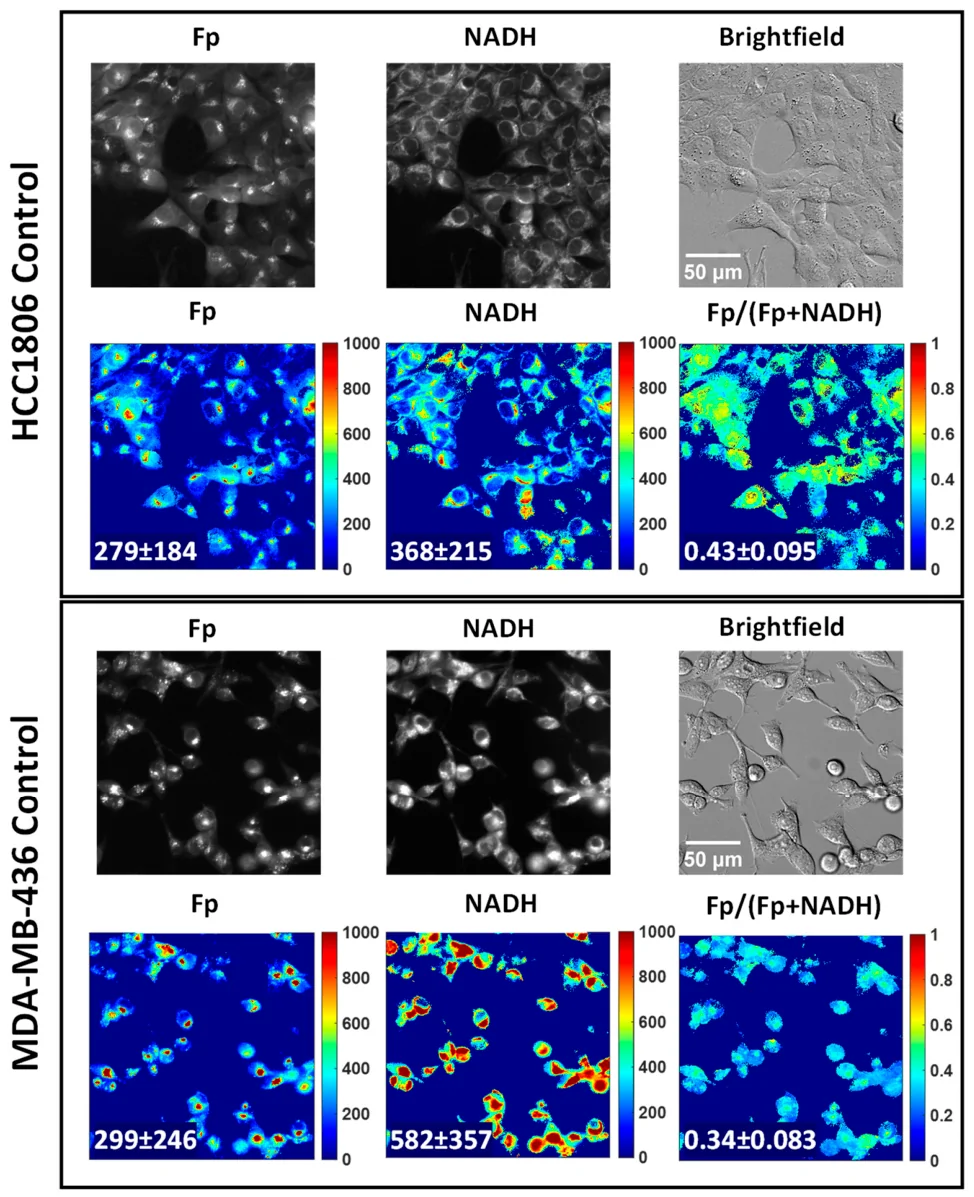
Representative raw, bright-field, and processed images of HCC1806 and MDA-MB-436 cells treated with 0.1% DMSO for 48 h. The nucleus appears as a dark, round area within the cell because both Fp and NADH signals are relatively low in the nucleus. The color bars for the Fp and NADH images indicate pixel intensities in arbitrary units, whereas those for the redox ratio images indicate normalized optical redox ratio (ORR = Fp/(NADH + Fp)) values ranging from 0 to 1. The values in the lower-left corners of the processed images indicate the mean ± SD of all pixels in the corresponding processed image.

**Figure 2:**
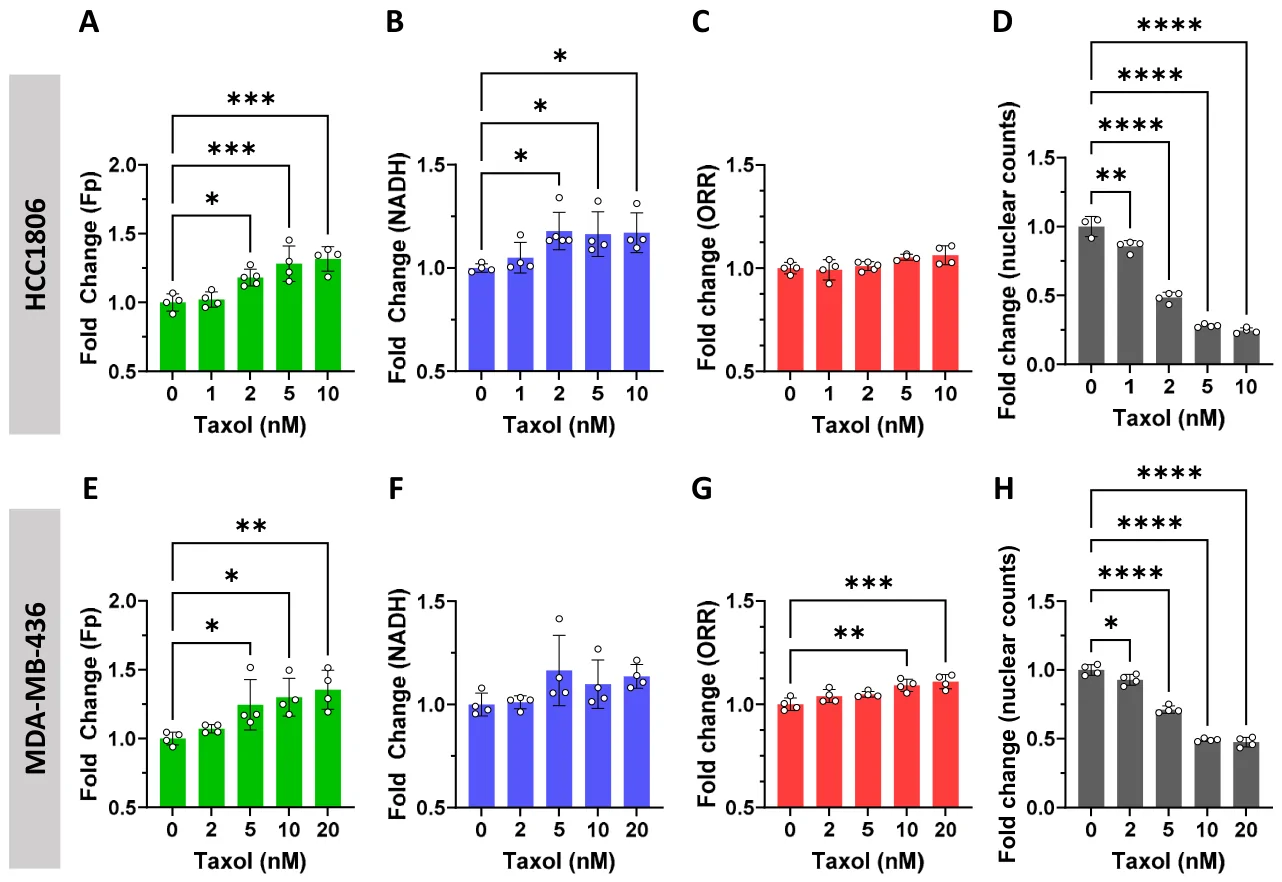
ORI responses and nuclear counts in HCC1806 (top) and MDA-MB-436 (bottom) cells following 48 h Taxol treatment. Fold changes in (**A**,**E**) Fp (green), (**B**,**F**) NADH (blue), (**C**,**G**) ORR (red), and (**D**,**H**) nuclear counts were quantified in HCC1806 (**A**–**D**) and MDA-MB-436 (**E**–**H**) cells. Nuclear counts were subsequently obtained by whole-dish Hoechst 33342 imaging of the same dishes used for ORI. Each open circle represents an individual dish. Statistical significance was determined by one-way ANOVA followed by Dunnett’s multiple comparisons test. *, *p* < 0.05; **, *p* < 0.01; ***, *p* < 0.001; ****, *p* < 0.0001.

**Figure 3:**
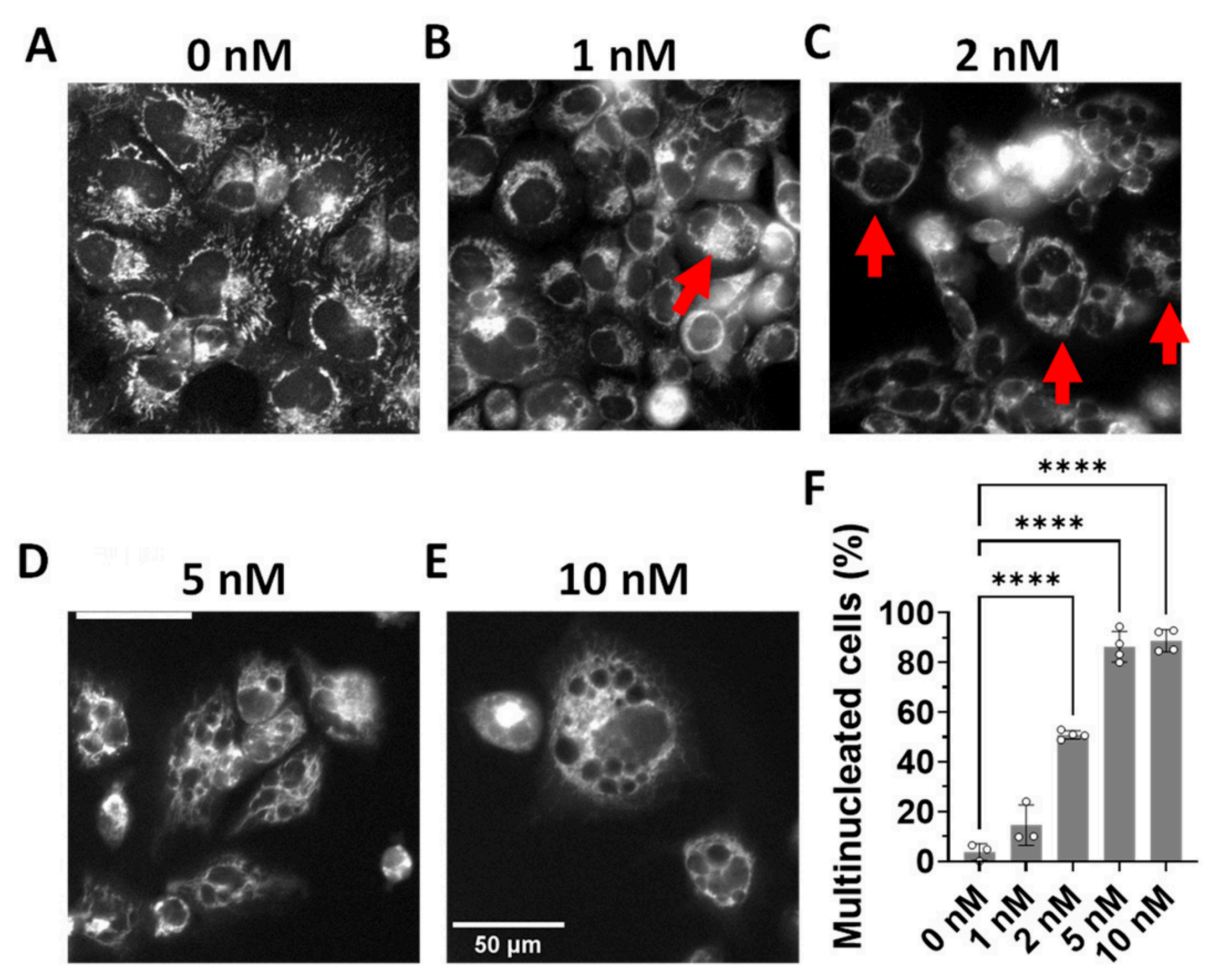
Taxol induces dose-dependent multinucleation in HCC1806 cells. (**A**–**E**) Representative mitochondrial NADH images of cells treated with DMSO, 1, 2, 5, or 10 nM Taxol, illustrating the increase in multinucleated cells with treatment, where red arrows point to typical multinucleated cells of various sizes. (**F**) The percentage of multinucleated cells quantified from NADH images. Bars represent mean ± SD, with each open circle representing an individual dish. ****, *p* < 0.0001.

**Figure 4:**
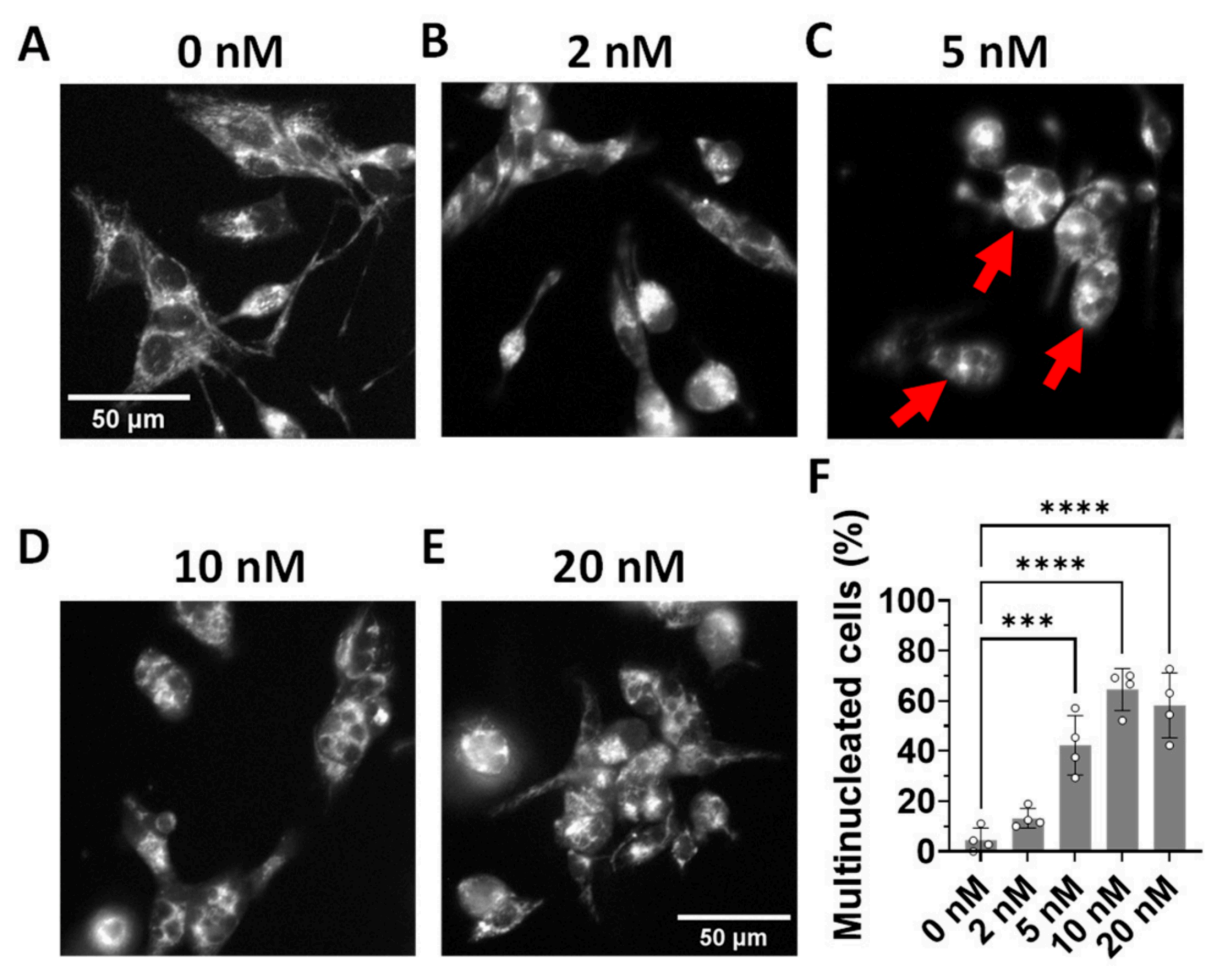
Taxol induces dose-dependent multinucleation in MDA-MB-436 cells. (**A**–**E**) Representative mitochondrial NADH images of cells treated with DMSO, 2, 5, 10, or 20 nM Taxol, illustrating the increase in multinucleated cells with treatment, where red arrows point to typical multinucleated cells of various sizes. (**F**) The percentage of multinucleated cells quantified from NADH images. Bars represent mean ± SD, with each open circle representing an individual dish. ***, *p* < 0.001; ****, *p* < 0.0001.

**Figure 5:**
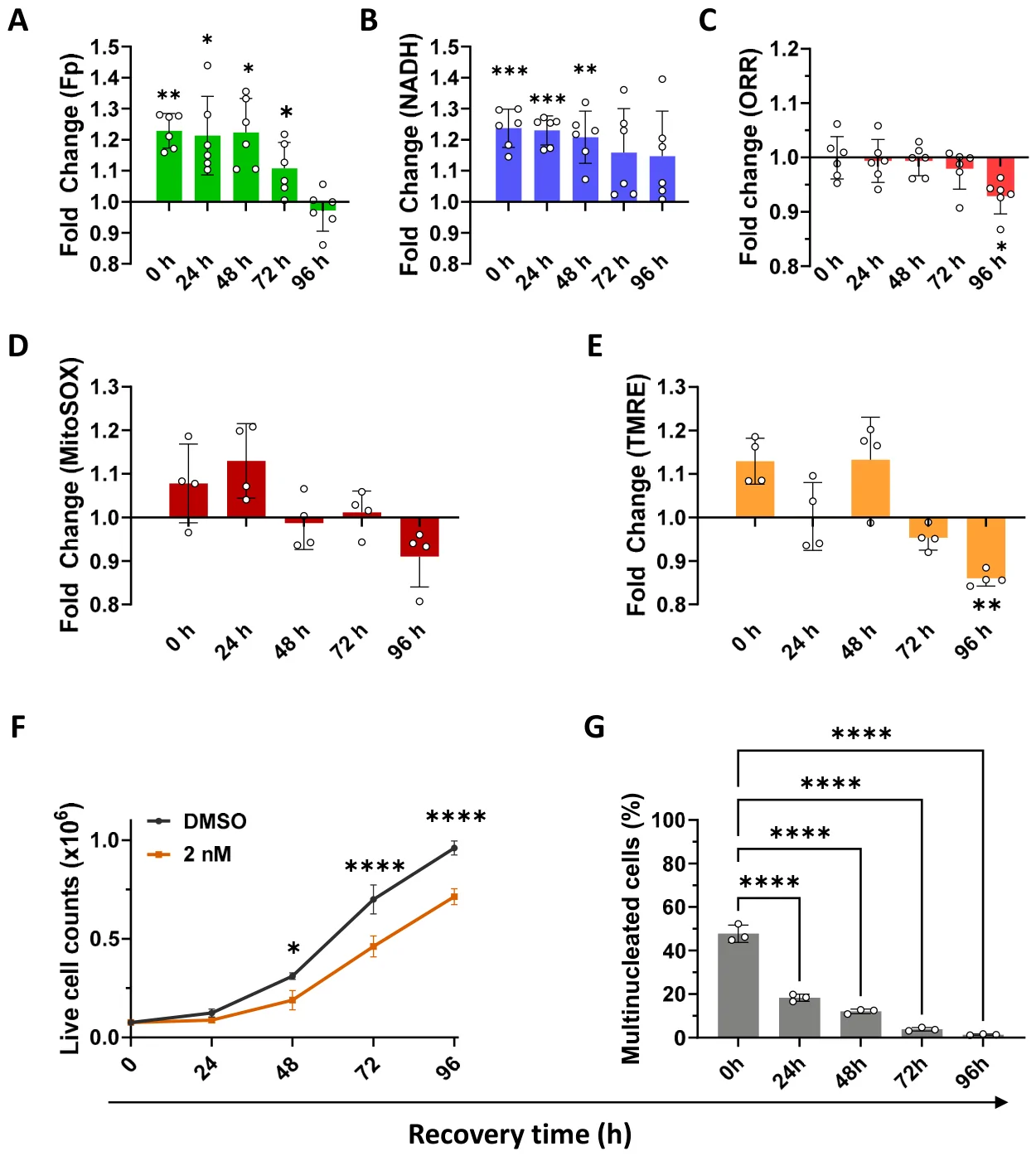
Recovery dynamics of mitochondrial redox status, mitochondrial function, cell population, and multinucleation following Taxol treatment in HCC1806 cells. HCC1806 cells were treated with 2 nM Taxol for 48 h, followed by recovery for 0, 24, 48, 72, or 96 h after Taxol removal, with 0 h representing the time point immediately after Taxol removal. Fold changes in (**A**) Fp (green), (**B**) NADH (blue), (**C**) ORR (red), (**D**) mito-ROS (MitoSOX, dark red), and (**E**) mitochondrial membrane potential (TMRE, orange). (**F**) Live-cell counts in 0.1% DMSO control and Taxol-pretreated cultures during recovery. (**G**) Percentage of multinucleated cells quantified from NADH images. For (**A**–**E**), statistical significance relative to the respective time-matched control (fold change = 1) was determined by two-tailed one-sample Student’s *t*-tests with Holm–Šídák correction across recovery time points. For (**F**), ordinary two-way ANOVA showed a significant treatment × recovery time interaction (F(3,16) = 8.45, *p* = 0.0014), followed by Šídák’s multiple comparisons test between DMSO- and Taxol-pretreated groups at each time point. For (**G**), statistical significance was determined by one-way ANOVA followed by Dunnett’s multiple comparisons test versus 0 h. Bars in (**A**–**E**,**G**) and symbols/lines in (**F**) represent mean ± SD. Each open circle represents an individual dish. For (**F**), *n* = 3 wells per group at each time point. *, adjusted *p* < 0.05; **, adjusted *p* < 0.01; ***, adjusted *p* < 0.001; ****, adjusted *p* < 0.0001.

**Figure 6:**
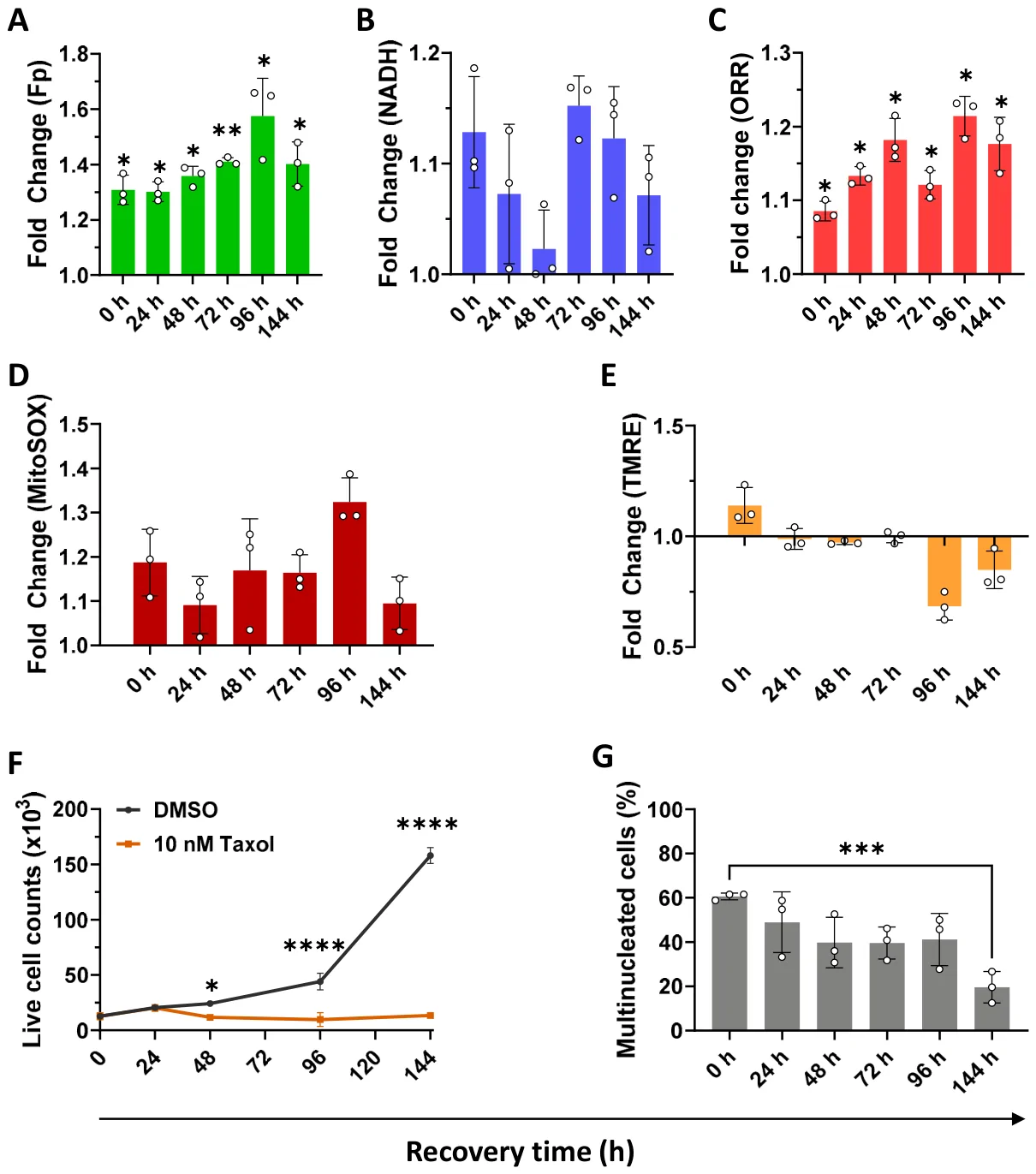
Recovery dynamics of mitochondrial redox status, mitochondrial function, cell population, and multinucleation following Taxol treatment in MDA-MB-436 cells. MDA-MB-436 cells were treated with 10 nM Taxol for 48 h, followed by recovery for 0, 24, 48, 72, 96, or 144 h after drug removal, where 0 h represents the time point immediately after Taxol removal. Fold changes in (**A**) Fp (green), (**B**) NADH (blue), (**C**) ORR (red), (**D**) mito-ROS (MitoSOX, dark red), and (**E**) mitochondrial membrane potential (TMRE, orange). (**F**) Live-cell counts in 0.1% DMSO control and Taxol-pretreated cultures during recovery. (**G**) Percentage of multinucleated cells quantified from NADH images. For (**A**–**E**), statistical significance relative to the respective time-matched control (fold change = 1) was determined by two-tailed one-sample Student’s *t*-tests with Holm–Šídák correction across recovery time points. For (**F**), ordinary two-way ANOVA showed a significant treatment × recovery time interaction (F(3,15) = 298.7, *p* < 0.0001), followed by Šídák’s multiple comparisons test between DMSO- and Taxol-pretreated groups at each time point. For (**G**), statistical significance was determined by one-way ANOVA followed by Dunnett’s multiple comparisons test versus 0 h. Bars in (**A**–**E**,**G**) and symbols/lines in (**F**) represent mean ± SD. Each open circle in (**A**–**E**,**G**) represents an individual dish. For (**F**), *n* = 3 wells per group at each time point, except for the DMSO group at 48 h (*n* = 2). *, adjusted *p* < 0.05; **, adjusted *p* < 0.01; ***, adjusted *p* < 0.001; ****, adjusted *p* < 0.0001.

**Figure 7:**
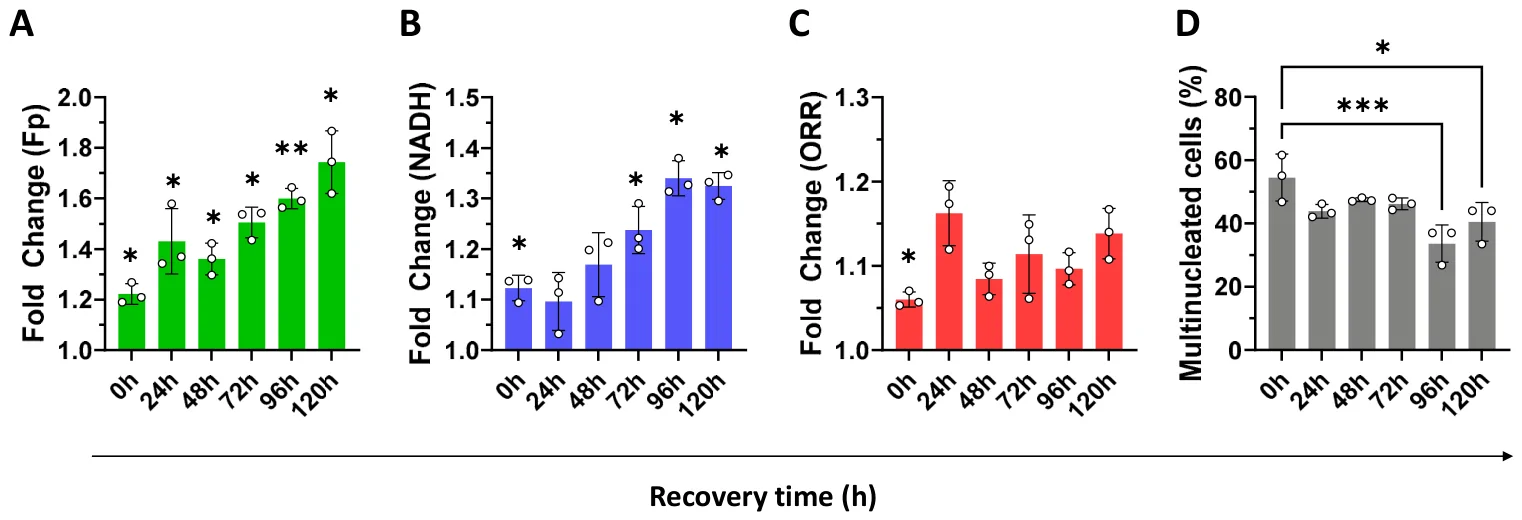
Recovery dynamics of mitochondrial redox status and multinucleation following removal of 20 nM Taxol in MDA-MB-436 cells. Fold changes in (**A**) Fp (green), (**B**) NADH (blue), and (**C**) ORR (red). (**D**) Percentage of multinucleated cells quantified from NADH images. For (**A**–**C**), statistical significance relative to the respective time-matched control (fold change = 1) was determined by two-tailed one-sample Student’s *t*-tests with Holm–Šídák correction across recovery time points. For (**D**), statistical significance was determined by one-way ANOVA followed by Dunnett’s multiple comparisons test versus 0 h. Bars represent mean ± SD. Each open circle represents an individual dish. *, adjusted *p* < 0.05; **, adjusted *p* < 0.01; ***, adjusted *p* < 0.001.

**Table 1:** The Zeiss microscope settings for all imaging sessions.

Channel	Excitation (nm)	Emission (nm)	LED Power	Exposure (s)	Gain	Incubation (min)
NADH	370–400	414–450	25%	2	1	N/A
Fp	450–488	500–530	40%	3	1	N/A
TMRE	540–570	580–610	40%	0.05	1	20
MitoSOX Red	370–400	580–610	40%	0.2	1	10

## Data Availability

The relevant data will be provided upon reasonable request.

## Supplementary Materials

The following supporting information can be downloaded at: https://www.mdpi.com/article/10.3390/cells15191752/s1.
