# Supplementary material for: Distinct Mitochondrial Recovery Dynamics Following Taxol Removal in Triple-Negative Breast Cancer Cells
Source: Cells. 2026 Sep 26;15(19):1752. doi: 10.3390/cells15191752 (PMC13664323; doi:10.3390/cells15191752)
Supplement: Supplementary file 1 [file cells-15-01752-s001.zip › cells-4461850-supplementary.pdf]

Supplementary materials

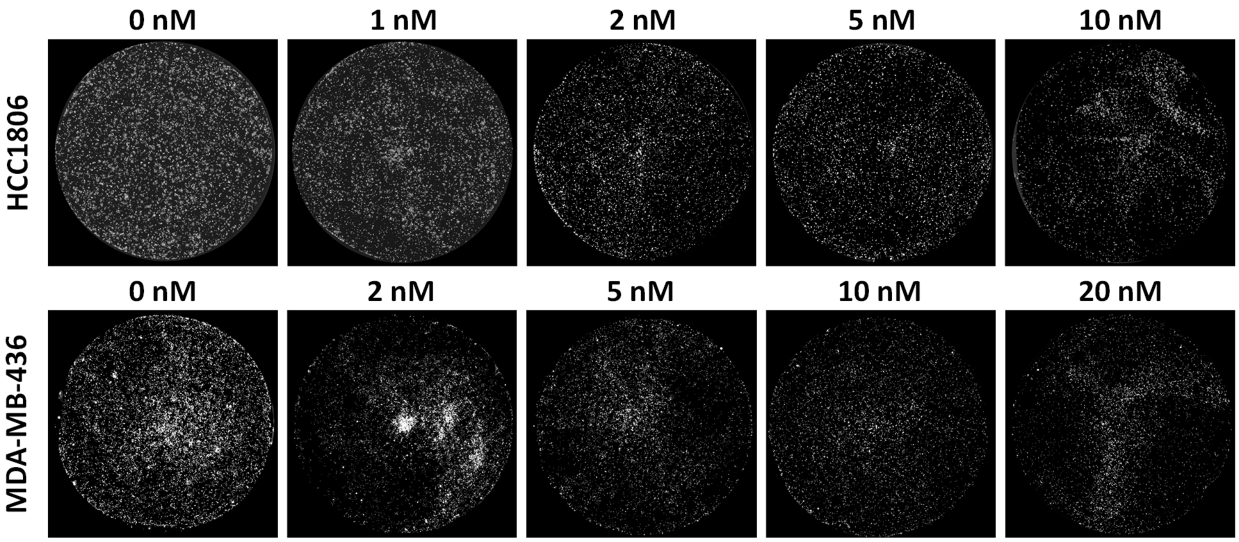

Figure S1. Representative whole-dish images of Hoechst 33342-stained nuclei in HCC1806 (top) and MDA-MB-436 (bottom) cells following 48 h Taxol treatment. Taxol concentrations are indicated above each image.

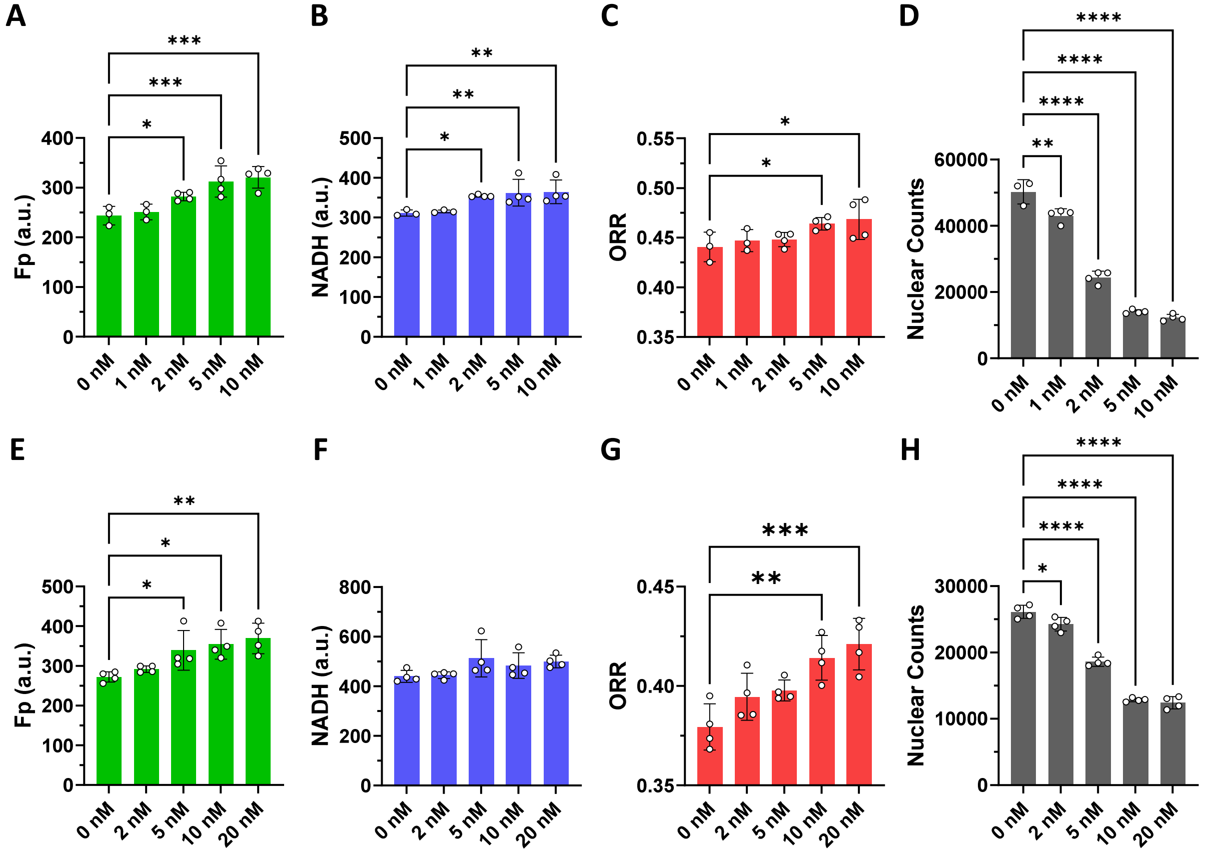

Figure S2. Absolute Fp, NADH, ORR, and nuclear-count measurements in HCC1806 (top) and MDA-MB-436 (bottom) cells at various Taxol concentrations. The x-axis indicates Taxol concentrations. Each open circle represents an individual dish. *, p<0.05; **, p<0.01; ***, p<0.001; ****, p<0.0001.

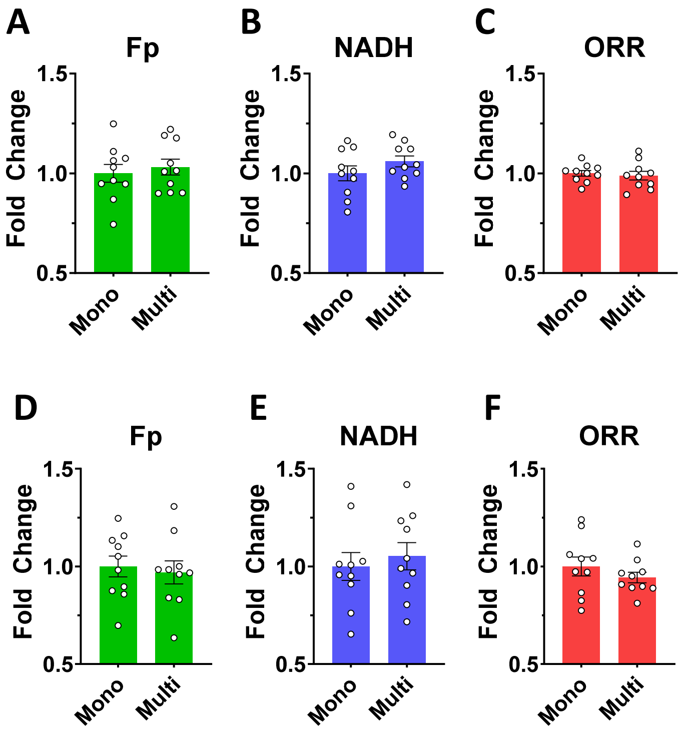

Figure S3. The redox indices of individual mononucleated and multinucleated cells showing similar values between the two cell populations. (A-C) HCC1806 treated with 2 nM Taxol for 48 h. (D-F) MDA-MB-436 cells treated with 20 nM Taxol for 48 h. The redox indices were quantified by manually drawing ROIs around them using NADH images. Each open circle represents an FOV.

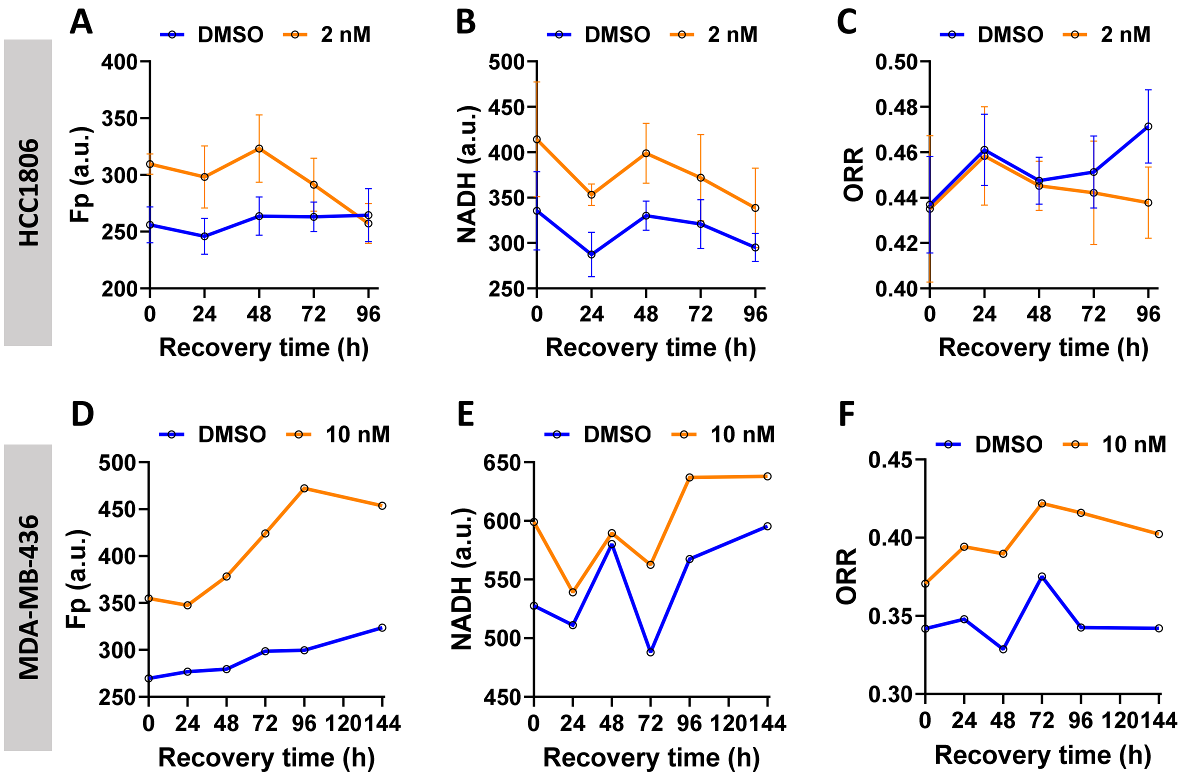

Figure S4. Absolute Fp, NADH, and ORR values during recovery in HCC1806 cells following 2 nM Taxol treatment (top) and MDA-MB-436 cells following 10 nM Taxol treatment (bottom). Cells were treated with Taxol for 48 h before drug withdrawal.

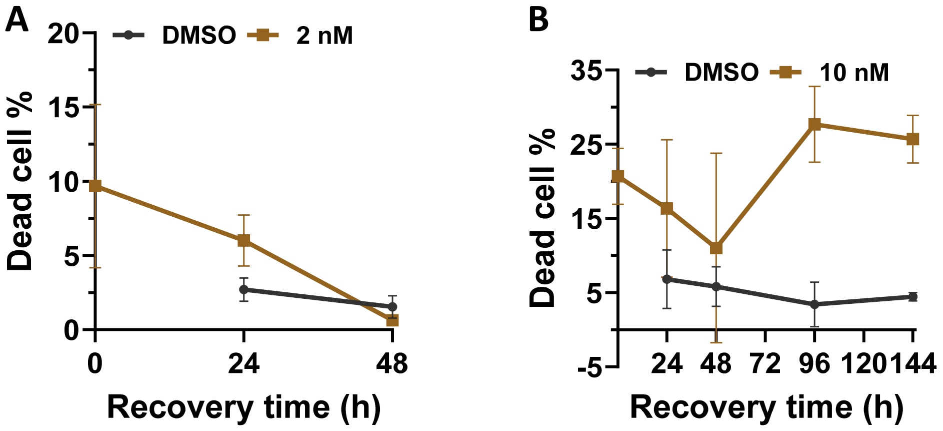

Figure S5. Percentage of dead cells (trypan blue-positive cells) during the recovery period for HCC1806 (A) and MDA-MB-436 (B) cultures.

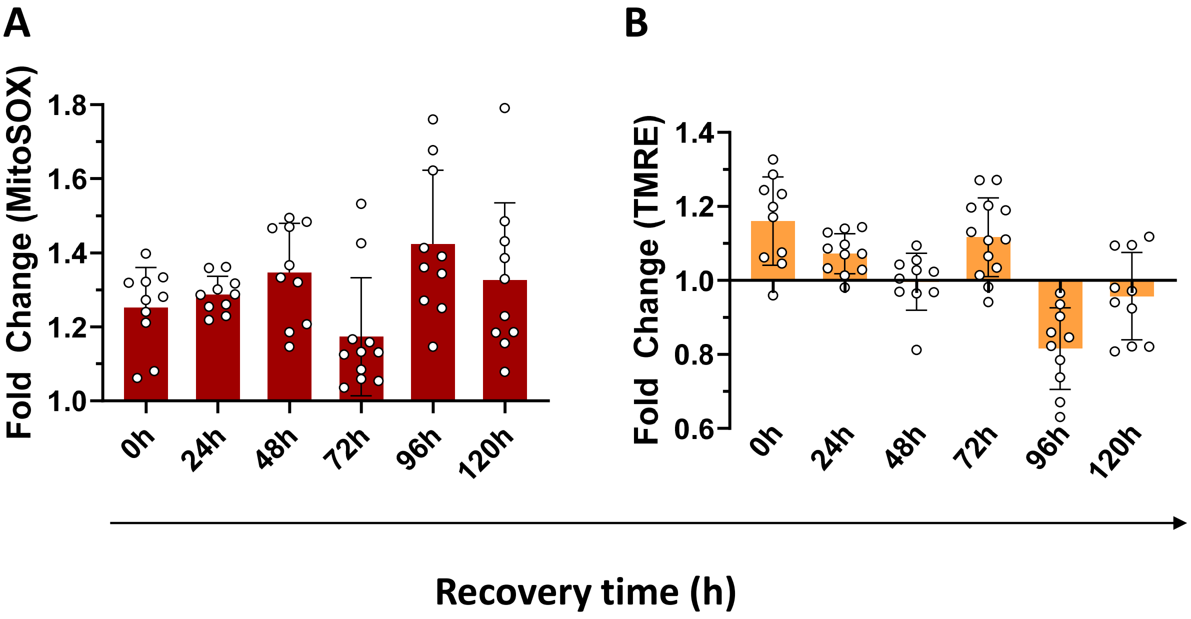

Figure S6. Mito-ROS and mitochondrial membrane potential during recovery of MDA-MB-436 cells following removal of 20 nM Taxol. (A) MitoSOX; (B) MMP. The readouts were normalized to the respective controls at each time point. Data are presented descriptively, with each open circle representing an FOV from the same dish at the specified recovery time.
